# Clinical features and relapse risks of IgG4-related ophthalmic disease: a single-center experience in China

**DOI:** 10.1186/s13075-021-02489-9

**Published:** 2021-03-31

**Authors:** Zhen Zhao, Dapeng Mou, Ziqiao Wang, Qiaozhu Zeng, Zhenfan Wang, Jimeng Xue, Limin Ren, Yanying Liu, Yin Su

**Affiliations:** 1 Department of Rheumatology and Immunology, Peking University People’s Hospital, 11 Xizhimen South Street, Beijing, 100044 China; 2 Beijing Tongren Eye Center, Beijing Ophthalmology & Visual Science Key Lab, Beijing Tongren Hospital, Capital Medical University, Beijing, 100730 China

**Keywords:** IgG4-related ophthalmic disease, Clinical features, Phenotypic differences, Relapse risks

## Abstract

**Background:**

IgG4-related ophthalmic disease (IgG4-ROD) is one of the phenotypes of IgG4-related disease (IgG4-RD), and its lesions are mainly located in the ocular. Currently, there are few studies on IgG4-ROD and no study has compared the phenotypic differences between IgG4-ROD and non IgG4-ROD (nIgG4-ROD). Thus, it is difficult to establish the optimal treatment strategy for IgG4-ROD. The aim of this study was to identify the disparities between the two groups and to clarify the risk factors for IgG4-ROD relapse.

**Methods:**

434 IgG4-RD patients met comprehensive diagnostic criteria and diagnosed at Peking University People’s Hospital between January 2009 and January 2020 were recruited in this study. Patients were divided into IgG4-ROD and nIgG4-ROD group according to the ophthalmic involvement. Demographic, clinical, and laboratory data of two groups were collected and compared. Cox regression analysis was used to identify the independent risk factors for IgG4-ROD relapse.

**Results:**

255 IgG4-ROD patients were identified in this study. IgG4-ROD group had almost equal sex ratio, younger age of disease onset and diagnosis comparing with nIgG4-ROD patients. As compared to nIgG4-ROD group, higher percentage of IgG4-ROD patients met the 2019 American College of Rheumatology/European League Against Rheumatism classification criteria (AECC) for IgG4-RD; moreover, IgG4-ROD patients had higher AECC scores and IgG4-RD responder index (RI). Allergic diseases and multiorgan involvement were more common in IgG4-ROD group. IgG4-ROD was frequently associated with salivary gland, paranasal sinus, lung, and lymph node involvement, while retroperitoneal fibrosis and biliary system lesions were more common in nIgG4-ROD. IgG4-ROD patients had higher serum IgG4 levels, IgG4/IgG ratio, IgE levels, and lower CRP levels. The initial glucocorticoid plus immunosuppressant was a protective factor for IgG4-ROD relapse. IgG4-ROD patients treated with initial glucocorticoid plus immunosuppressant had longer relapse-free survival time than patients treated with initial glucocorticoid monotherapy.

**Conclusions:**

IgG4-ROD patients had distinctive clinical features compared with nIgG4-ROD patients. The initial glucocorticoid plus immunosuppressant was a protective factor for IgG4-ROD relapse, which could prolong the relapse-free survival time of IgG4-ROD patients. These findings may have important implications for understanding and management of IgG4-ROD.

## Background

IgG4-related disease (IgG4-RD) is a new clinicopathological entity, originally proposed by Kamisawa et al. in 2003 [1]. Although the exact etiology and pathogenesis remain largely unknown, remarkable progress has been made in the understanding of this disease. Now it has been recognized as an immune-mediated systemic fibroinflammatory disease featuring tumefactive lesions [2]. Serum IgG4 level is elevated in the majority of patients with IgG4-RD, but this is a nonspecific finding [3]. Characteristic histopathologic features are lymphoplasmacytic infiltration rich in IgG4-immunopositive plasma cells, storiform fibrosis, and obliterative phlebitis [4, 5]. IgG4-RD is a highly heterogenous disorder that can affect almost every organ system in the body [6–9], and the ocular lesions are also extremely common.

In the past, different terms have been used to name IgG4-RD with ocular lesions [10–14]. However, with the publication of comprehensive diagnostic criteria for IgG4-RD in 2012 [15], IgG4-related ophthalmic disease (IgG4-ROD) has been regarded as a standardized name for IgG4-RD with ophthalmic involvement. The ocular anatomic structures affected in IgG4-ROD include lacrimal gland, soft tissue, extraocular muscle, eyelid, optical nerve, orbital bone, sclera, conjunctiva, and trigeminal nerve, and extraocular involvement can also occur with ocular lesions simultaneously or metachronously [16]. We define these cases who fulfill comprehensive diagnostic criteria for IgG4-RD but have no ocular lesions as non IgG4-ROD (nIgG4-ROD). So IgG4-RD can be divided into IgG4-ROD and nIgG4-ROD according to whether there is ophthalmic involvement. Currently, there has been no study to explore the differences in clinical features between IgG4-ROD and nIgG4-ROD patients.

The use of systemic glucocorticoids as first-line therapy for IgG4-ROD usually leads to an effective initial response [17]. However, the relapse rate of IgG4-ROD is quite high during glucocorticoid tapering or withdrawal [18]. Therefore, prolonged the use of glucocorticoids is necessary to prevent relapse, but it often leads to many steroid-related adverse events. Immunosuppressants or biological agent rituximab as a second or third-line treatment has been effectively used in the treatment of recurrent or refractory IgG4-ROD patients [17, 18]. However, the optimal treatment strategy for IgG4-ROD has not yet been established.

In this study, we focused on comparing the clinical features between IgG4-ROD and nIgG4-ROD patients and exploring the risk factors of IgG4-ROD patients. The aim was to identify the disparities between the two groups and to clarify the risk factors for IgG4-ROD relapse, so as to further improve the understanding of IgG4-RD with different phenotypes and help to better formulate diagnosis and management strategies.

## Methods

### Study population

This retrospective study was conducted in the Peking University People’s Hospital, a total of 434 patients with IgG4-RD who have attended at our hospital between January 2009 and January 2020 were recruited. Patients with infections, tumors, or other autoimmune diseases were excluded from this study. All enrolled patients met the IgG4-RD comprehensive diagnostic criteria [15]: (1) clinical examination shows characteristic diffuse/localized swelling or masses in single or multiple organs, (2) hematological examination shows elevated serum IgG4 concentrations (> 135 mg/dl), and (3) histopathologic examination shows (a) marked lymphocyte and plasmocyte infiltration and fibrosis or (b) infiltration of IgG4+ plasma cells (ratio of IgG4+/IgG+ cells > 40% and > 10 IgG4+ plasma cells/high power field). Whether the clinical symptoms, signs, physical examinations, or imaging tests including ultrasound, computed tomography (CT), magnetic resonance imaging (MRI) or positron emission tomography/computed tomography (PET/CT) revealed ophthalmic involvement in a patient with IgG4-RD, the patient was considered to have IgG4-ROD. Patients were divided into IgG4-ROD and nIgG4-ROD group according to the ophthalmic involvement. This study was approved by the Medical Ethics Committee of Peking University People’s Hospital (Beijing, China).

### Data collection

All patients’ clinical data including age, sex, history of allergic disease, initial lesion site, number of organs involved, and ophthalmic symptoms were collected from medical records. IgG4-RD responder index (RI) [19] and 2019 American College of Rheumatology/European League Against Rheumatism classification criteria (AECC) for IgG4-RD [20] scores were calculated. The diagnosis of allergic disease was made by specialists based on the criteria of the European Academy of Allergy and Clinical Immunology. The laboratory parameters including complete blood count, liver function, serum IgG, serum IgG4, serum IgE, antinuclear antibody (ANA), rheumatoid factor (RF), erythrocyte sedimentation rate (ESR), C-reactive protein (CRP), and complement tests were analyzed retrospectively. Different imaging tests such as ultrasound, CT, MRI, or PET/CT were performed in all patients to evaluate the organ involvement. Totally, 240 patients underwent tissue biopsy, and the samples were fixed with formaldehyde solution, routine paraffin embedding, and hematoxylin-eosin staining. Immunohistochemical staining was performed using antibodies to IgG and IgG4. The pathological diagnosis of IgG4-RD was confirmed by the experienced pathologists after ruling out all potential alternate diagnoses. The initial medication regimens, duration of immunosuppressants, and the relapse and relapse-free survival time during follow-up period of IgG4-ROD patients were reviewed. Data regarding patients’ clinical features of the two groups were compared. Cox regression analysis was used to identify the independent risk factors for IgG4-ROD relapse.

### Statistical analysis

The quantitative variables were described as mean ± standard deviation (SD) or median (interquartile range, IQR). Categorical variables were presented as proportions. Differences between groups were analyzed using two independent samples *t* test or Wilcoxon rank sum test for quantitative variables, and chi-square test or Fisher’s exact test for categorical variables. Univariate and multivariate Cox regression analyses (Enter method) were performed to identity the possible risk factors for IgG4-ROD relapse. Variables with *P* < 0.2 in the univariate analysis were included in the multivariate analysis. The Kaplan-Meier survival curve and log-rank test were used to compare the difference of the initial treatment options on the relapse of IgG4-ROD. All the data were processed by the IBM SPSS Statistics version 23.0 software. Significant differences were defined as *P* < 0.05.

## Results

### Demographic characteristics

Altogether, 434 patients with IgG4-RD were recruited in this study including 247 males and 187 females. Respectively, 212 (48.8%), 28 (6.5%), and 194 (44.7%) patients were diagnosed as definite, probable, and possible IgG4-RD, according to the IgG4-RD comprehensive diagnostic criteria. Among these patients, 255 cases were identified with IgG4-ROD and another 179 cases were diagnosed with nIgG4-ROD. The demographic characteristics of the patients are displayed in Table 1. We could observe several differences between IgG4-ROD and nIgG4-ROD patients. The sex ratio of male to female in the IgG4-ROD group was approximately equal (0.96:1), lower than that in nIgG4-ROD group (2.14:1) (*P* < 0.001). The mean age of disease onset in IgG4-ROD group and nIgG4-ROD group was 51.1 ± 13.2 years and 55.5 ± 13.5 years, respectively, suggesting the former had an earlier disease onset (*P* = 0.001). The mean age of disease diagnosis in IgG4-ROD group was 54.4 ± 12.6 years, which was younger than that in nIgG4-ROD group, but there was no statistical difference between the two groups (Table 1).

**Table 1:** Demographic and clinical features of the patients

Characteristics	IgG4-RD	IgG4-ROD	nIgG4-ROD	*P* value
Number of cases, *n*	434	255	179	
Male to female	1.32:1	0.96:1	2.14:1	< 0.001*
AECC score	33.0 ± 14.2	37.0 ± 12.9	27.2 ± 14.0	< 0.001*
AECC score ≥ 20, *n* (%)	344(79.3)	228(89.4)	116(64.8)	< 0.001*
Age of disease onset (years), mean ± SD	52.9 ± 13.5	51.1 ± 13.2	55.5 ± 13.5	0.001*
Age of disease diagnosis (years), mean ± SD	55.4 ± 12.9	54.4 ± 12.6	56.9 ± 13.4	0.054
History of allergic disease, *n* (%)	214(49.3)	167(65.5)	47(26.3)	< 0.001*
IgG4-RD RI	11.0 ± 6.2	11.4 ± 6.0	10.2 ± 6.3	0.045*
Number of involved organs, median (IQR)	5(3–6)	6(4–7)	3(2–5)	< 0.001*
Number of involved organs, *n* (%)				
1 organ involved	30(6.9)	4(1.6)	26(14.5)	< 0.001*
2 organs involved	52(12.0)	11(4.3)	41(22.9)	< 0.001*
3 organs involved	46(10.6)	20(7.8)	26(14.5)	0.026*
≥ 4 organs involved	306(70.5)	220(86.3)	86(48.0)	< 0.001*

The *P* value refers to the comparison between IgG4-ROD and nIgG4-ROD patients. *AECC* American College of Rheumatology/European League Against Rheumatism classification criteria, *IgG4-RD RI* IgG4-RD responder index, *SD* standard deviation, *IQR* interquartile range. *P < 0.05

### Clinical features

**Fig. 1:**
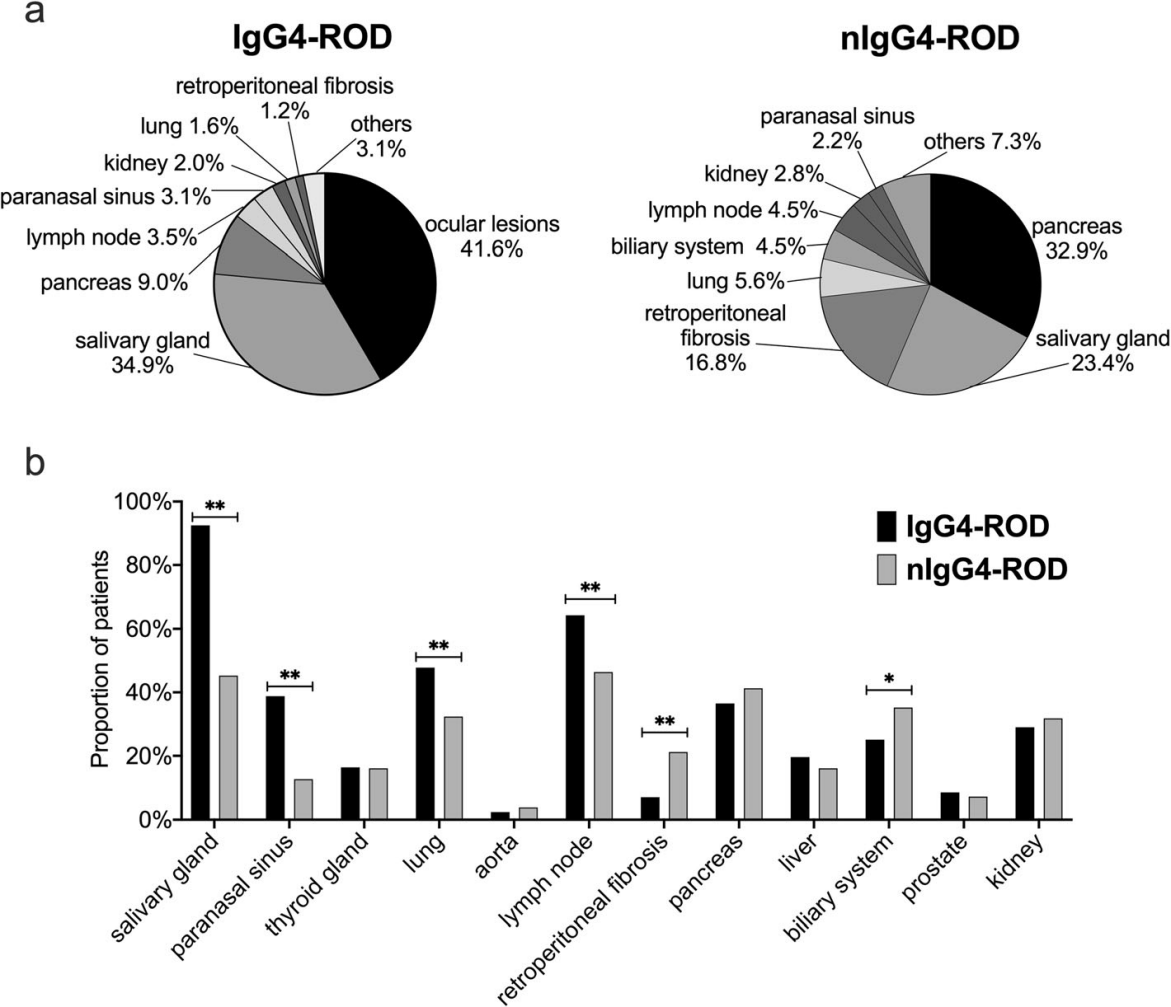
The initial lesion site and organ involvement of patients with IgG4-ROD and nIgG4-ROD. **a** The constituent ratio of initial lesion site in IgG4-ROD and nIgG4-ROD patients. The three most frequent initial lesions in IgG4-ROD patients were the ocular (41.6%), salivary gland (34.9%), and pancreas (9.0%), while in nIgG4-ROD patients were the pancreas (32.9%), salivary gland (23.4%), and retroperitoneal fibrosis (16.8%). **b** Comparison of organ involvement except for ocular between patients with IgG4-ROD and nIgG4-ROD. The percentages of patients with the salivary gland, paranasal sinus, lung, and lymph node involvement in IgG4-ROD group were higher than those in nIgG4-ROD group, while nIgG4-ROD patients had higher percentages of retroperitoneal fibrosis and biliary system lesions. *P < 0.05, **P < 0.01

As compared to nIgG4-ROD group, higher percentage of IgG4-ROD patients met the 2019 AECC for IgG4-RD (*P* < 0.001); moreover, IgG4-ROD patients had higher AECC scores (*P* < 0.001) and IgG4-RD RI (*P* = 0.045). Notably, the prevalence of allergic diseases (including allergic rhinitis, asthma, and urticaria) in IgG4-ROD group was higher than that in nIgG4-ROD group (*P* < 0.001) (Table 1). The initial lesion sites at disease onset of two groups are shown in Fig. 1a. There was a significant difference in the constituent ratio of the initial lesion site between the two groups. It was found that the three most common initial lesion sites in IgG4-ROD patients were the ocular (41.6%), salivary gland (34.9%), and pancreas (9.0%), while those in nIgG4-ROD patients were the pancreas (32.9%), salivary gland (23.4%), and retroperitoneal fibrosis (16.8%).

In this study, we observed that a single organ involvement was very rare, and most patients (93.1%) had multiple organ involvement. By comparison, it was found that the median number of involved organs and the percentage of ≥ 4 organs involved in IgG4-ROD group were significantly higher than those in nIgG4-ROD group (Table 1). The involvement of other organs except for ocular is displayed in Fig. 1b. After comparison, we found that the percentages of patients with the salivary gland, paranasal sinus, lung, and lymph node involvement in IgG4-ROD group were higher than those in nIgG4-ROD group, while nIgG4-ROD patients had higher percentages of retroperitoneal fibrosis and biliary system lesions.

**Fig. 2:**
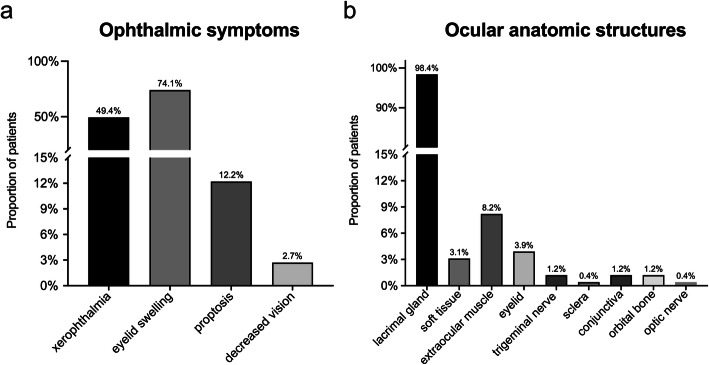
Rate of ophthalmic symptoms and affected ocular anatomic structures in IgG4-ROD patients. **a** Proportions of patients with different ophthalmic symptoms in IgG4-ROD patients. The most frequent ophthalmic symptom was eyelid swelling (74.1%), followed by xeropthalmia (49.4%) and proptosis (12.2%), while decrease vision (2.7%) was relatively rare. **b** Proportions of patients with different ocular anatomic structures involved in IgG4-ROD patients. The lacrimal gland (98.4%) occupied the most frequently affected ocular anatomic structure, followed by the extraocular muscle (8.2%), while the eyelid (3.9%), soft tissue (3.1%), conjunctiva (1.2%), trigeminal nerve (1.2%), orbital bone (1.2%), sclera (0.4%), and optic nerve (0.4%) were relatively rare.

We also analyzed the ophthalmic symptoms and the affected ocular anatomic structures of IgG4-ROD patients (Fig. 2). Eyelid swelling (74.1%) was the most frequent ophthalmic symptom, followed by xeropthalmia (49.4%) and proptosis (12.2%), while decrease vision (2.7%) was relatively rare (Fig. 2a). The affected ocular anatomic structure in IgG4-ROD patients was evaluated in detail. Highly consistent with the ophthalmic symptoms, the lacrimal gland (98.4%) occupied the most frequent affected ocular anatomic structure in IgG4-ROD patients, followed by extraocular muscle (8.2%), while the eyelid (3.9%), soft tissue (3.1%), conjunctiva (1.2%), trigeminal nerve (1.2%), orbital bone (1.2%), sclera (0.4%), and optic nerve (0.4%) were relatively rare (Fig. 2b).

### Laboratory parameters

The laboratory parameters of the two groups are summarized in Table 2. There were statistical differences in IgG4, IgG4/IgG ratio, IgE, and CRP between two groups. IgG4-ROD patients had higher serum IgG4 levels (*P* < 0.001) and IgG4/IgG ratio (*P* < 0.001) than nIgG4-ROD patients. IgG4-ROD group also had higher serum IgE levels and the higher proportion of patients with elevated IgE than nIgG4-ROD group (*P* = 0.022 and *P* = 0.008, respectively). While nIgG4-ROD patients had higher CRP levels and the higher proportion of patients with elevated CRP (*P* = 0.004 and *P* = 0.001, respectively). In addition, higher ESR levels were also found in nIgG4-ROD group, but there was no statistical difference between two groups. In our study, no significant differences were found in the eosinophilia, complement, globulin, IgG, ANA, and RF results between the two groups.

**Table 2:** Comparison of laboratory parameters between patients with IgG4-ROD and nIgG4-ROD

Laboratory parameter	IgG4-ROD	nIgG4-ROD	*P* value
Low serum C3, *n* (%)	87(39.7)	51(34.7)	0.330
Low serum C4, *n* (%)	75(34.7)	44(29.9)	0.340
Hypocomplementemia, *n* (%)	100(45.9)	63(42.9)	0.570
Elevated ESR, *n* (%)	94(49.5)	78(55.3)	0.293
Elevated CRP, *n* (%)	30(16.3)	45(31.9)	0.001*
Elevated globulin, *n* (%)	54(28.6)	41(29.1)	0.920
Eosinophilia, *n* (%)	60(27.8)	33(21.6)	0.176
RF+, *n* (%)	43(20.5)	26(18.7)	0.684
ANA+, *n* (%)	32(14.3)	25(16.7)	0.542
Elevated IgE, *n* (%)	178(84.0)	77(71.3)	0.008*
C3 (g/L), mean ± SD	0.852 ± 0.280	0.935 ± 0.319	0.053
C4 (g/L), mean ± SD	0.184 ± 0.095	0.200 ± 0.093	0.115
ESR (mm/h), median (IQR)	18.5(8.0–40.0)	23.5(10.0–59.0)	0.058
CRP (mg/L), median (IQR)	2.24(1.00–4.70)	3.05(1.26–12.45)	0.004*
Globulin (g/L), median (IQR)	33.7(29.7–41.5)	35.4(29.8–41.5)	0.808
Serum IgG4 (mg/dl), median (IQR)	983(409–1838)	523(225–1370)	< 0.001*
Serum IgG (g/L), median (IQR)	17.5(14.3–23.2)	17.2(14.1–22.0)	0.609
IgG4/IgG, median (IQR)	0.529(0.272–0.879)	0.294(0.143–0.629)	< 0.001*
Serum IgE (IU/ml), median (IQR)	339.7(160.8–847.6)	259.4(83.1–653.3)	0.022*

*SD* standard deviation, *IQR* interquartile range. *P < 0.05

### Treatment and relapse of IgG4-ROD

**Fig. 3:**
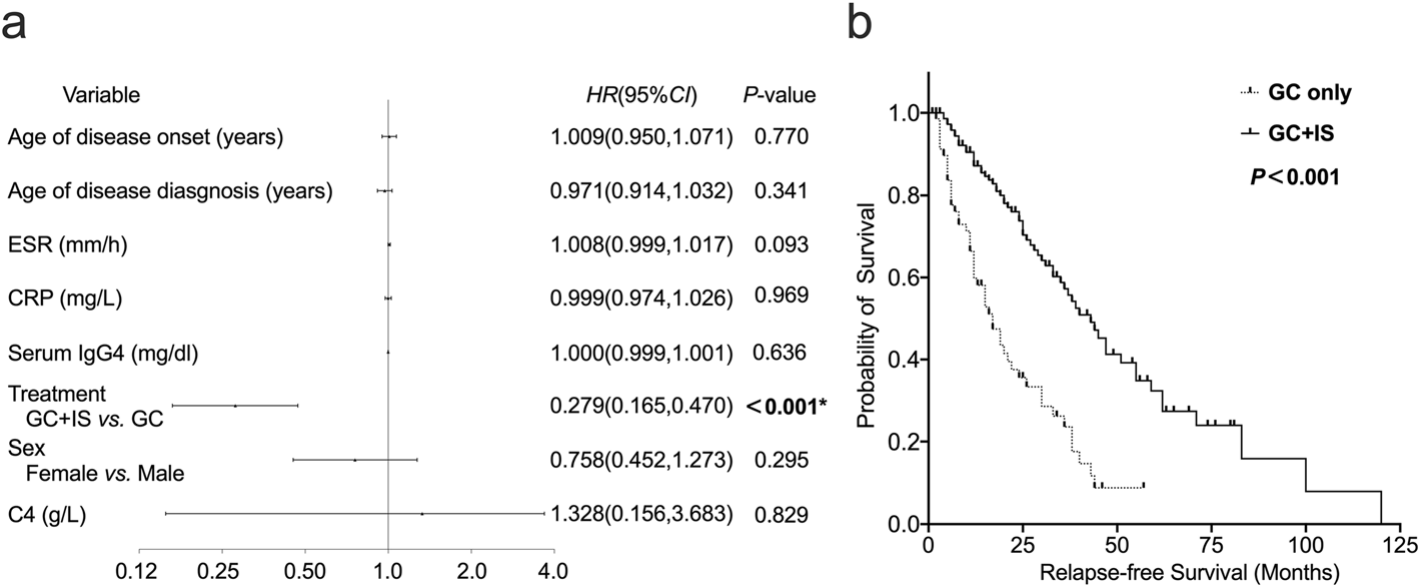
Forest plot of multivariate Cox regression analysis and Kaplan-Meier curve for IgG4-ROD relapse. **a** Forest plot of multivariate Cox regression analysis for IgG4-ROD relapse. The initial glucocorticoid plus immunosuppressant was a protective factor for IgG4-ROD relapse (*P* < 0.001). **b** Kaplan-Meier curve of relapse-free survival of IgG4-ROD patients based on the initial treatment options. IgG4-ROD patients treated with initial GC only vs IgG4-ROD patients treated with initial GC + IS (*P* < 0.001). HR hazard ratio, CI confidence interval, GC glucocorticoid, IS immunosuppressant. *P < 0.05

Univariate Cox regression analysis revealed that sex, age of disease onset, and age of disease diagnosis; levels of serum C4, ESR, CRP, and IgG4; and initial treatment options were associated with IgG4-ROD relapse (Table 3). Multivariate Cox regression analysis identified initial glucocorticoid plus immunosuppressant was a protective factor for IgG4-ROD relapse (Fig. 3a). The Kaplan-Meier curves of relapse-free survival of IgG4-ROD patients based on the initial treatment options were plotted in Fig. 3b. According to the initial treatment options, IgG4-ROD patients were divided into initial glucocorticoid monotherapy group and initial glucocorticoid plus immunosuppressant therapy group. Oral glucocorticoids were given at a dose of 0.6–0.8 mg/kg/day in both groups and then tapered by 10 mg every 2–4 weeks. Immunosuppressants were selected according to the severity of the disease, patient’s tolerance, and economic affordability, including cyclophosphamide, mycophenolate mofetil, methotrexate, leflunomide, azathioprine, iguratimod, and rituximab. The median duration of immunosuppressants was 21 (8–36) months. Respectively, the median relapse-free survival times for the initial glucocorticoid monotherapy group and the initial glucocorticoid plus immunosuppressant group were 17 months and 43 months, and the latter group had significantly longer relapse-free survival time. In addition, the log-rank test revealed that there was significant difference between the two relapse-free survival curves, the initial glucocorticoid plus immunosuppressant group had an obviously higher relapse-free survival rate than the initial glucocorticoid monotherapy group (*P* < 0.001).

**Table 3:** Univariate Cox regression analysis for IgG4-ROD relapse

Variable	Univariate analysis		
	*HR*	95%*CI*	*P* value
Sex			
Male	1		Ref
Female	0.777	0.534, 1.130	**0.187**^**#**^
Age of disease onset (years)	0.989	0.974, 1.004	**0.141**^**#**^
Age of disease diagnosis (years)	0.986	0.971, 1.001	**0.059**^**#**^
History of allergic disease			
No	1		Ref
Yes	1.133	0.754, 1.701	0.548
Number of involved organs, *n*	1.050	0.965, 1.143	0.258
C3 (g/L)	1.489	0.668, 3.319	0.330
C4 (g/L)	0.218	0.023, 2.089	**0.187**^**#**^
ESR (mm/h)	1.009	1.001, 1.017	**0.035**^**#**^
CRP (mg/L)	1.015	0.995, 1.034	**0.140**^**#**^
Serum IgG4 (mg/dl)	1.000	0.999, 1.001	**0.147**^**#**^
Serum IgE (IU/ml)	1.000	0.999, 1.001	0.772
Eosinophilia			
No	1		Ref
Yes	1.031	0.663, 1.604	0.892
Treatment			
GC only	1		Ref
GC + IS	0.332	0.224, 0.491	**< 0.001**^**#**^

*HR* hazard ratio, *CI* confidence interval, *Ref* reference, *GC* glucocorticoid, *IS* immunosuppressant. ^**#**^*P* < 0.2

## Discussion

IgG4-RD is an immune-mediated systemic fibroinflammatory disease featuring tumefactive lesions, which can affect almost every organ system in the body. IgG4-ROD refers to IgG4-RD with ophthalmic manifestations and is one of the phenotypes of IgG4-RD. There are currently few studies focusing on IgG4-ROD, and no research has explored the differences in the clinical features between IgG4-ROD and nIgG4-ROD. Thus, it is difficult to establish the best treatment strategy for IgG4-ROD. Therefore, we conducted a large retrospective cohort study to compare demographic characteristics, clinical presentations, and laboratory parameters between IgG4-ROD patients and nIgG4-ROD patients. In addition, the risk factors for IgG4-ROD relapse were also identified.

Currently, there is no exact data on the epidemiology of IgG4-ROD due to the lack of well recognition and wide understanding of IgG4-ROD. Several previous studies conducted in Japanese and US populations have reported that the frequency of IgG4-ROD ranges 4 to 34% of IgG4-RD patients [21–24]. While the frequency of IgG4-ROD in our cohort accounted for 58.8% of IgG4-RD patients. Maybe racial variations, sample size, or admission unit contribute to the difference of the frequency of IgG4-ROD in IgG4-RD.

Our research showed that IgG4-ROD patients had almost equal sex ratio, younger age of disease onset and diagnosis, and higher IgG4-RD RI. In addition, the initial lesion sites in majority of IgG4-ROD patients were mainly located in the ocular (41.6%) and salivary gland (34.9%), which were directly exposed to the external environment and lack barrier protection. The damage of tissue and immune tolerance to autoantigen in genetically susceptible individuals after exposure to certain environmental factors (allergens, bacteria, etc.) is considered to be a plausible mode of pathogenesis of IgG4-RD [2]. The reason why IgG4-ROD patients develop symptoms and get diagnosis at an earlier age, and have almost equal sex ratio may be that barrier-deficient organs can be exposed to the environmental triggers more easily, earlier, and with equal chance for both sexes.

IgG4-RD RI as a tool to evaluate the disease activity of IgG4-RD was used in this study. The higher IgG4-RD RI in IgG4-ROD group suggested that IgG4-ROD may have higher disease activity and may require more aggressive treatment. PET/CT is also considered to facilitate the management of IgG4-ROD, due to its good performance in detecting organ involvement, differentiating active and inactive lesions, and monitoring treatment response and relapse during IgG4-RD diagnosis and follow-up [25, 26]; however, it is not covered by medical insurance in China—most of the patients did PET/CT to exclude tumor in our cohort.

With regard to the affected ocular anatomic structure in IgG4-ROD, we found that the lacrimal gland and extraocular muscle were most frequently involved, while other anatomic structures were rarely involved, which is highly in line with previous researches [16, 17]. Similarly, eyelid swelling and xerophthalmia caused by lacrimal gland involvement were the most frequent manifestations in IgG4-ROD. More than 90% (≥ 2 organs involved) of IgG4-ROD patients had extraocular manifestations, and the most common sites of extraocular involvement were the salivary gland and lymph node, which is similar with the previous study [27]. In addition, our study showed that IgG4-ROD patients had more organs involvement. Therefore, it is recommended that systemic examination and imaging evaluation should be carried out in IgG4-ROD patients to identify other organ involvement as early as possible and start systematic treatment.

In terms of laboratory parameters, IgG4-ROD patients had lower levels of ESR and CRP, which may reflect the absence of a higher activity of acute inflammation in IgG4-ROD. As we all know, elevated serum IgG4 concentration is one of the hallmark features of IgG4-RD. Previous studies have confirmed that serum IgG4 tends to be higher in IgG4-RD patients with the lacrimal or salivary gland [21, 28] and multiple organ involvement [14, 29]. We also found that higher serum IgG4 levels and the IgG4/IgG ratio were found in IgG4-ROD patients in our study. Moreover, IgG4-ROD patients had higher serum IgE levels, higher frequencies of elevated IgE, and allergic disease.

It is currently believed that abnormal immune response against infection, allergen, or tissue injury may be the initiating factors in the pathogenesis of IgG4-RD, and then triggers can activate T follicular helper (Tfh) and T follicular regulatory (Tfr) cell immune response, and the subsequent production of IL-4/IL-10 cytokines can promote the production and class switch of IgG4 and IgE in plasmablasts, eosinophil recruitment, and fibroblast activation [2, 30, 31] Finally, it leads to the onset of IgG4-RD. Recent studies have identified that CD4+ cytotoxic T lymphocytes (CTLs) may also play a central role in this pathophysiological process [32, 33]. It is well known that in some autoimmune diseases such as systemic lupus erythematosus, the emergence of certain autoantibodies is often associated with the heterogeneity in organ involvement and clinical manifestations [34]. IgG4-RD is thought to have similar profiles with the identification of diverse autoantigens that may serve as potential triggers [35–38]. Therefore, we suppose that differences in local allergen or autoantigen exposure may result in the different clinical and serological features of the two IgG4-RD phenotypes in this study. However, the exact mechanism remains unclear and needs further study.

The phenotypic differences of IgG4-RD have been more clearly understood after IgG4-RD was classified into four different groups: pancreato-biliary (group 1), retroperitoneum/aortitis (group 2), head and neck limited (group 3), and Mikulicz/systemic (group 4) [39]. When groups 3 and 4 are analyzed together, they can largely represent IgG4-ROD in this study. Compared with the results of IgG4-ROD in our study, group 3 plus 4 in the study with 42.2% of Asians [39] also has more organ involvement, ocular, salivary gland, paranasal sinus, lung, and lymph node involvement and higher serum IgG4 levels, but lower male to female sex ratio and retroperitoneal and biliary system lesions. However, another study with 95% of Caucasians seems to have higher levels of IgG4 and IgE in group 1 [40]. Therefore, possible genetic or ethnic factors also contribute to the clinical and serological differences between IgG4-ROD and nIgG4-ROD.

There are currently no clinical treatment guidelines to instruct the diagnosis and treatment of IgG4-ROD. The use of systemic glucocorticoids as first-line treatment for majority of IgG4-ROD patients usually results in the improvement of symptoms and decrease of serum IgG4 levels; however, the relapse is not uncommon during glucocorticoid tapering or withdrawal [17, 18]. In this study, we found that initial glucocorticoid plus immunosuppressant was a protective factor for IgG4-ROD relapse. IgG4-ROD patients initially treated with glucocorticoid plus immunosuppressant had longer relapse-free survival time compared with the initial glucocorticoid monotherapy group. In our previous study, although there was the same trend as this one, we did not find a statistically significant correlation between the initial glucocorticoids plus immunosuppressants and the relapse of IgG4-RD [41]. The reason may be that the population and variables included in the two studies are different. Therefore, large cohort, prospective randomized controlled trials are still needed to evaluate the value of glucocorticoid plus immunosuppressants in the treatment of IgG4-ROD in future.

Our study has some limitations. First, our study suffers from the limitation of its retrospective design, non-randomized controlled investigation, and treatment protocol. Second, this study was conducted in a single rheumatology center, thus possibly leading to a selection bias that cases with multiple organ involvements were reported.

## Conclusions

In conclusion, our study demonstrated and confirmed on the largest cohort of IgG4-ROD patients that IgG4-ROD showed younger age of disease onset and diagnosis, and almost equal sex ratio. IgG4-ROD group had higher AECC scores and IgG4-RD RI. Allergic diseases and multiorgan involvement were more frequent in IgG4-ROD group. Moreover, IgG4-ROD patients had higher serum IgG4 levels, IgG4/IgG ratio, and serum IgE levels. The salivary gland, paranasal sinus, lung, and lymph node involvement were more frequent in IgG4-ROD patients. In addition, initial glucocorticoid plus immunosuppressant was a protective factor, which could prolong the relapse-free survival time of IgG4-ROD patients. These findings may have important implications for the understanding and management of IgG4-ROD.

## Data Availability

The datasets used and/or analyzed during the current study are available from the corresponding author on reasonable request.

## Abbreviations

AECC: American College of Rheumatology/European League Against Rheumatism Classification Criteria
ANA: Antinuclear antibody
C3: Complement 3
C4: Complement 4
CI: Confidence interval
CRP: C-reactive protein
CT: Computed tomography
CTLs: Cytotoxic T lymphocytes
ESR: Erythrocyte sedimentation rate
GC: Glucocorticoid
HR: Hazard ratio
IgE: Immunoglobulin E
IgG: Immunoglobulin G
IgG4: Immunoglobulin G4
IgG4-RD: IgG4-related disease
IgG4-RD RI: IgG4-RD responder index
IgG4-ROD: IgG4-related ophthalmic disease
IQR: Interquartile range
IS: Immunosuppressant
MRI: Magnetic resonance imaging
nIgG4-ROD: Non- IgG4-related ophthalmic disease
PET/CT: Positron emission tomography/computed tomography
Ref: Reference
RF: Rheumatoid factor
SD: Standard deviation
Tfh: T follicular helper
Tfr: T follicular regulatory
